# A role for the asexual spores in infection of *Fraxinus excelsior* by the ash-dieback fungus *Hymenoscyphus fraxineus*

**DOI:** 10.1038/srep34638

**Published:** 2016-10-03

**Authors:** Helen Nicola Fones, Charlotte Mardon, Sarah Jane Gurr

**Affiliations:** 1 Department of Biosciences, University of Exeter, Stocker Road, Exeter, EX4 4QD, UK; 2 Rothamsted Research, North Wyke Farm Platform, Okehampton, Devon, EX20 2SB, UK.

## Abstract

The invasive pathogen, ash dieback fungus *Hymenoscyphus fraxineus*, is spreading rapidly across Europe. It shows high levels of outcrossing and limited population structure, even at the epidemic front. The anamorphic (asexual) form produces prolific conidia, thought to function solely as spermatia (male gametes), facilitating gene flow between sympatric strains. Here, we show that conidia are capable of germination on ash leaves and *in vitro*, and can infect seedlings *via* leaves or soil. In leaves, germlings form structures resembling fruiting bodies. Additionally, *H. fraxineus* colonises ash debris and grows in soil in the absence of ash tissues. We propose an amended life-cycle in which wind-dispersed, insect-vectored or water-spread conidia infect ash and may sporulate *in planta,* as well as in forest debris. This amplifies inoculum levels of different strains in ash stands. In combination with their function as spermatia, conidia thus act to maximise gene flow between sympatric strains, including those originally present at low inoculum. Such mixing increases evolutionary potential, as well as enhancing the likelihood of gene introgression from closely-related strains or assimilation of further genetic diversity from parental Asian populations. This scenario increases the adaptability of *H. fraxineus* to new climates and, indeed, onto new host species.

The ascomycete fungus *Hymenoscyphus fraxineus*^1^ is the causal agent of ash dieback^2^. This disease is currently destroying ash trees across Europe^3^,^4^,^5^. First observed in Poland in 1992^3^, it has now spread to more than 25 European countries^6^. This pathogen is aggressive^7^, causing leaf necrosis, premature leaf drop, shoot wilting and crown dieback, as well as necrotic lesions on petioles, stems and root collars^7^. Mortality is high in infected seedlings, whilst older trees develop chronic infections which are often eventually fatal (e.g. ref. 8). The loss of trees has a significant ecological and economic impact^9^, as ash is an important tree of mature woodland and hedgerows, as well as yielding commercial timber^10^,^11^.

*Hymenoscyphus fraxineus* is pleiomorphic; the anamorph (previously *Chalara fraxinea*^12^) produces prolific asexual spores^13^,^14^. However, these conidia have not been thought to have a role in the spread of the fungus, being described as “sticky”^3^,^15^ and unlikely to become airborne. They are instead assumed to act as spermatia^13^,^16^ during ascospore formation by the teleomorph (previously *H. pseudoalbidus*^17^). This hypothesis is supported by the discovery that ascospores derived from individual apothecia show levels of genetic polymorphism indicative of multiple mating partners^13^,^14^ and by the observation that the anamorph sporulates at the edge of the pseudosclerotial plate from which the apothecia emerge^18^.

Evidence suggests that sexual reproduction is of key importance the spread of *H. fraxineus*, a heterothallic fungus^13^. Population genetic studies have found high intra-population variability, along with little inter-population variability^19^ and a lack of population genetic structure^14^,^20^,^21^,^22^,^23^,^24^ across the entire continent of Europe. These data indicate high gene flow between populations, consistent with a high rate of outcrossing^14^. Indeed, while clear evidence of a founder effect can be seen when comparing the genotypes of the invasive pathogen populations in Europe with the much more variable genotypes displayed by native *H. fraxineus* populations in Asia, no such founder effect has been detected in the study of newly-discovered populations at the epidemic front^19^. The closely-related species, *Hymenoscyphus albidus*, a non-pathogenic saprophyte on ash debris^25^,^26^, is being displaced and is facing local extirpation in regions colonised by *H. fraxineus*^27^,^28^. *H. albidus* is homothallic and lacks an anamorphic stage^29^. As such, the invasive *H. fraxineus* may have an advantage over *H. albidus*, due to a greater adaptive potential.

The current best model for the disease cycle of *H. fraxineus* proposes that ash leaves are infected by wind-dispersed ascospores^5^,^13^,^15^. These spores are known to travel up to 50 metres^15^ and their long distance dispersal over kilometres is inferred from population genetics^13^. Ascospores germinate on leaves and invade the vascular tissue^2^,^30^. Some hyphae progress into the stem via the petiole, causing bark lesions, although this is relatively rare^31^. Infected leaves drop to the forest floor in autumn, where pseudosclerotial plates form on rachises and petioles, allowing the pathogen to overwinter^7^,^25^,^27^,^32^. In spring, apothecia emerge on the previous years’ rachises, and ascospores are released once more^32^.

It is, however, remarkable that the anamorph produces prolific numbers of conidia both in axenic culture and on infected rachises. The conidia are thought to be transported in water between different strains infecting the same rachis, and between different rachises on the forest floor^13^,^33^. The purpose of this energetically-demanding profusion of conidia remains unclear, considering that they travel only short distances and are produced among abundant mating partners. Recently, microconidia of the ascomycete rice blast fungus, *Magnaporthe oryzae*, similarly believed to function exclusively as spermatia, were found to germinate on both artificial and leaf surfaces, and to cause disease in rice and barley^34^. The germination rate was low, at 5–10%, of which germlings only 17% were viable^34^. Given this overall viability of only 0.85–1.7% for microconidia, it is unsurprising that their function in disease had been overlooked.

Here, we test the hypothesis that the *H. fraxineus* conidia germinate at low frequency and cause occasional infection, like the microconidia of *M. oryzae*. We therefore tested the ability of these asexual spores to: i. germinate *in vitro* and *in planta*; ii. infect young ash seedlings and iii. initiate saprophytic growth on ash debris (seed coats and fallen leaves) and in soil, with a view to eliminating or elucidating their potential roles in the disease biology of *H. fraxineus*.

## Methods

### Strains and culture conditions

*H. fraxineus* strain HP11 (kindly provided by Dr J Webber, Forest Research, UK) was cultured on Malt Extract or V8 Agar plates and incubated at 18 °C in natural lighting conditions. Strains were stored on agar slopes at 4 °C. Extensive, synchronous sporulation was induced by transferring four week old cultures to 4 °C and maintaining them in the dark for two weeks.

### Ash seedling culture

Ash *(Fraxinus exelsior)* seeds were obtained from Forestart Ltd (Shrewsbury, UK). They were stored at 4 °C until use and germinated in John Innes No. 2 soil in a growth cabinet with 16 h day length and day/night temperatures of 18 °C/14 °C. When seedlings developed their first true leaves, they were transferred to individual 5 cm soil pots. For experiments involving inoculated soil, seeds were germinated on trays containing the treated soil and then thinned to 24 seedlings per tray at the cotyledon stage. Seeds which failed to germinate were not removed, but left on the soil surface.

### Leaf and soil inoculations

Twelve week old seedlings were transferred to day/night temperatures of 16 °C/10 °C. For leaf inoculations, 5 ml of 0.1% (v/v) Tween 20 (in sterile distilled water) was added to sporulating cultures of *H. fraxineus* and agitated gently to create a spore suspension. Spore suspensions were passed twice through double-layers of Miracloth to remove any fragments of mycelium and visual checks made with a dissecting microscope to confirm that only spores were present. Suspensions were centrifuged for 5 min at 5000 g and the spore pellet re-suspended in 0.1% Tween 20 to give a final concentration of 10^7^ spores per millilitre. This was applied to both surfaces of all true leaves using a paintbrush. For soil inoculation, spore suspensions were obtained in the same way, but spore concentrations were not set by spore counts. Instead, the sporulating area of culture from which the spores were obtained was measured, using photography and simple image analysis in ImageJ^35^. ‘Low spore’ inoculum was harvested from one or the equivalent of one 10 cm^2^ petri dish. ‘High spore’ inoculum was derived from ten times that surface area. As positive controls, agar plates containing the same areas of culture were scraped to give a mixture of mycelium and spores. Inocula were mixed thoroughly with 500 g (dry weight) of John Innes No. 2 compost, on which ash seeds were subsequently germinated as described. For the soil growth experiments, mycelium was scraped from plates, weighed, and 1 g samples ground with 0.1 ml of sterile distilled water using an eppendorf pestle. Samples were diluted to 1 ml and added drop by drop to 25 g (dry weight) soil in a 10 cm^2^ petri dish. Inoculated soil was stirred thoroughly before and after addition of a further 10 ml water and incubation at room temperature.

### DNA extraction, PCR detection and qPCR quantification of *H. fraxineus*

DNA extraction was carried out on leaf samples using a modified phenol-chloroform extraction protocol (e.g. ref. 36). Samples were flash frozen and ground in liquid nitrogen, and incubated for 30 min at RT in 1 ml modified Tris-EDTA DNA extraction buffer containing 6% (v/v) SDS plus 0.5 ml of 0.1 mm diameter glass beads per sample. Incubation was combined with vibrational mixing at 2000 rpm using a Vibrax^TM^ orbital shaker. Root and ash debris samples were treated in the same manner as leaves, after removing adhering soil by extensive rinsing in sterile distilled water. DNA was extracted from soil samples using the protocol described by Yeates *et al*.^37^, replacing the final phenol-chloroform extraction step with the protocol described above. PCR detection of *H. fraxineus* in extracted DNA was performed using DreamTaq Green PCR Master Mix and 30 cycles of 30 s, 95 °C melting, 30 s, 60 °C annealing and 1 min, 72 °C extension with specific primers (fwd: CTTGCGATCGTTGTGGTTGTGAG, rev: TCGACCAGTTGTGTAAACTCCAC), kindly provided by Dr S Kilaru (University of Exeter). For quantitative real-time PCR of soil DNA samples, Soil and control HP11 DNA samples were diluted to 100 ng/μl in milliQ water, using a nanodrop^®^ spectrophotometer to measure DNA concentration. Control HP11 DNA was then serially diluted to give a ladder of *H. fraxineus* DNA concentrations (100, 50, 10, 5 and 1 ng/μl). Soil DNA samples were diluted to a final concentration of 50 ng/μl. qPCR was then carried out using SYBR green master mix (Thermo), 1 μl of template DNA and two primer sets, designed to the *glyceraldehyde-3-phosphate dehydrogenase (gpd)* (fwd: ATGGCTCCTACTAAGATTGGAATCA; rev: GCTCGATGAAGGGATCGTTGACA) and *α*-*tubulin* (fwd: ATGCGTGAAGTCATCAGCATCAAC; rev: TCTGTAGTCGTTGATGTATCTTACC) genes, using a Mx3005P qPCR System (Agilent) on the default SYBR green settings. Ladder samples and no-template controls were run twice, each time in quintuplicate and test samples once, in triplicate. Primer efficiencies were calculated from the slopes of the standard curves of Ct *vs* log_10_[DNA] (*gpd*–88.6%; *α*-*tubulin*–85.4) and the R^2^ values for the standard curves were 0.92 (*gpd*) and 0.90 (*α*-*tubulin*). Concentration of *H. pseudoalbididus* DNA in the total soil DNA samples was then calculated from the relevant standard curve.

### Germination assays on artificial surfaces

Ash leaves were immersed in chloroform for five minutes and 500 μl of the resulting chloroform/leaf wax solution dripped onto hydrophobic glass cover slips. After evaporation of the chloroform, these cover slips were inoculated with 10 μl of a freshly prepared 10^7^/ml spore suspension and incubated in a moist chamber at 18 °C for 24 h under natural lighting conditions.

### Tissue staining and confocal microscopy

Leaf and root samples were immersed for one hour, in the dark, in a solution of 0.05% (w/v) propidium iodide (PI) and 0.05% (w/v) fluorescein isothiocyanate (FITC) conjugated lectin (wheatgerm agglutinin, WGA), prior to confocal microscopy. For visualisation, samples were mounted in 0.1% (v/v) phosphate buffered saline (PBS, pH 7). Spores and germlings on artificial surfaces were stained with 5 μl each of PI and FITC, at the same concentrations as above. Confocal microscopy was carried out using argon laser emission at 500 nm with detection in 600–630 nm (PI, red) and 510–530 nm (FITC, green), using a Leica SP8 confocal microscope.

### Scanning Electron Microscopy of sporulating fungus

Sporulating fungus was visualised by SEM after 14 days incubation at 4 °C, and again after a further 14 days. A cryo-SEM procedure was used in which small samples of sporulating fungus were removed from the surface of agar plates and rapidly frozen in liquid nitrogen slush. Samples were then etched and sputter-coated for visualisation. This procedure was carried out using a Jeol JSM-6390LV cryo-SEM rig with Gatan Cryo-transfer system.

## Results

### Asexual spores of *H. fraxineus* germinate with low efficiency on artificial surfaces and on ash leaves

**Figure 1:**
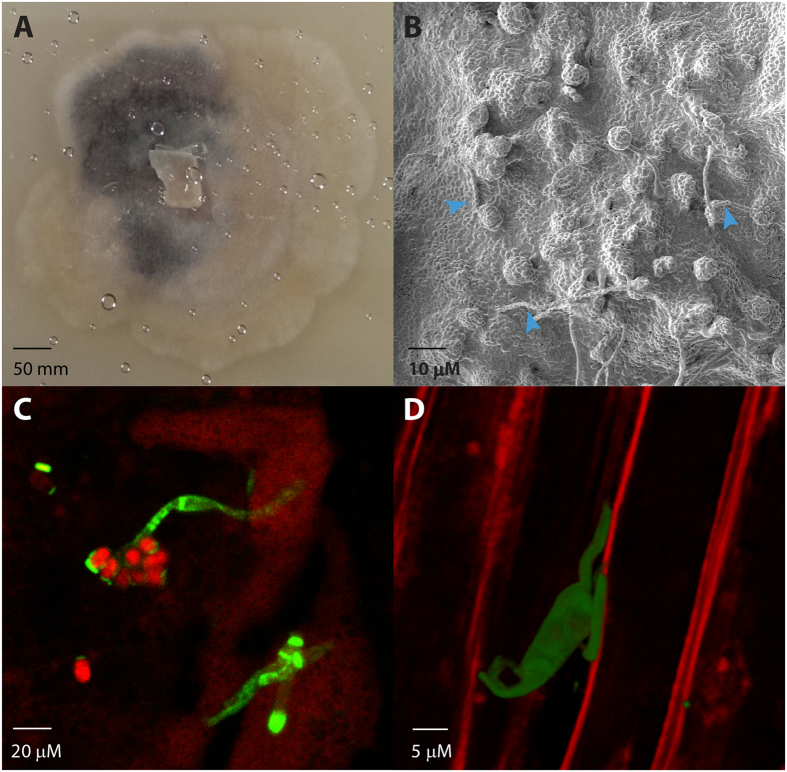
Asexual spores of *H. fraxineus*. (**A**) *H. fraxineus* produces asexual spores readily when cultured on MEA or V8 for 28 days at 18 °C followed by 14 days at 4 °C (HP11). (**B**) Asexual spores are abundant, appearing as a ‘sea’ in this scanning electron micrograph of strain HP11 on an MEA plate. Globules of spores and spore chains (arrows) can also be seen. (**C**) Asexual spores germinate on a hydrophobic glass slide overlaid with ash leaf waxes. (**D**) Asexual spores germinate on the ash leaf surface.

Asexual spores of *H. fraxineus* are produced readily and abundantly *in vitro* (Fig. 1A). Such axenic cultures produced both aerial spores (used throughout this work) and spores embedded into the agar itself. Visualisation of sporulating cultures two weeks after transfer to 4 °C (total age = 6 weeks) by SEM revealed droplets and chains of spores, as described previously^12^. Older cultures, visualised four weeks after transfer to 4 °C (total age = 8 weeks), appear as a ‘sea’ of spores in which these structures remain visible (Fig. 1B).

If conidia of *H. fraxineus* function in dispersal of the pathogen and in infection of ash, as well as their putative function as spermatia, then they must be able to germinate. To test this, we harvested conidia and inoculated hydrophobic glass slides, coated with ash leaf waxes, and also leaves of ash seedlings. After 24 h incubation on these surfaces, conidia/germlings were stained with FITC-conjugated WGA and visualised by confocal microscopy. Germination could be seen from one, or occasionally both, ends of the conidium (Fig. 1C,D). Co-staining with propidium iodide revealed that most of the non-germinating spores were non-viable (Fig. 1C). Germination was scored on three independently prepared artificial surfaces, and the numbers of germinated *vs*. non-germinated spores recorded (total number of spores scored per surface: 100). Germination frequency was determined to be 6.3 ± 0.9%. On leaf surfaces, spore germination was also observed (Fig. 1D), but again, this was rare.

### Ash seedlings inoculated with asexual spores of *H. fraxineus* show symptoms consistent with ash dieback disease

Following foliar inoculation with *H. fraxineus* ash seedlings remained symptomless for 7–10 days. After this period, small, necrotic lesions began to develop on the leaves, with browning of the leaf vasculature apparent and wilting of leaves and stems. After 14 days, some leaves became entirely necrotic. Necrotic leaves were subsequently abscised. Forty five percent of inoculated seedlings died within three weeks.

### *H. fraxineus* hyphae ramify over the leaf surface, associating primarily with the vasculature, and form putative sporulating structures within the leaf epidermis

**Figure 2:**
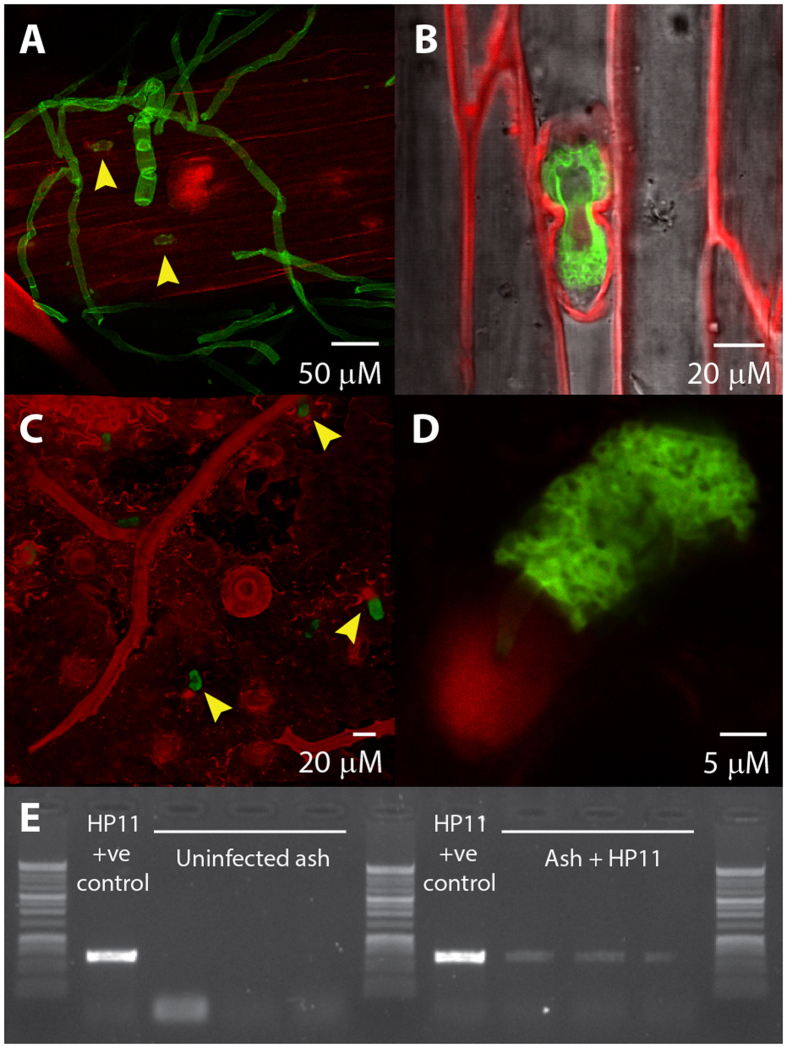
Ash leaves infected with asexual spores of *H. fraxineus*. Confocal microscopy images showing *H. fraxineus* stained with FITC-WGA (green) on ash leaves stained with PI (red). (**A**) *H. fraxineus* grows over the leaf surface after germination of asexual spores applied as foliar inoculum, associating particularly with the vasculature. Putative fungal fruiting bodies can also be seen within some vascular epidermal cells (yellow arrowheads). (**B**) Enlarged view showing one of these structures in more detail. (**C,D**) (enlarged): Similar structures also appear attached to the rest of the leaf epidermis. (**E**) PCR detection of *H. fraxineus* in ash seedlings inoculated with asexual spores *via* leaves (+ve control, *H. fraxineus* DNA from axenic cultures).

Leaves were sampled from infected, symptomatic seedlings at twelve days post inoculation and stained with PI and FITC-WGA. Confocal imaging of these samples revealed fungal hyphae growing over the leaf surface, associated particularly with the abaxial surfaces of the vascular tissues (Fig. 2A). Within the epidermal cells covering the vascular tissue, fungal inclusions can be seen. These are complex multi-cellular structures which appear to be surrounded by ectopic plant cell wall tissue (Fig. 2B). Similar structures can be seen emerging from, or attached to, the abaxial leaf surface away from the vascular tissue, with a ring of PI-stained tissue at the plant-fungus interface (Fig. 2C,D). These rings are not stained with FITC-WGA are thus likely to be of plant origin. The precise nature of the fungal structures remains elusive, but they are suggestive of *in planta* sporulation. Confirmation that the observed fungus is, indeed, *H. fraxineus*, was obtained by PCR amplification of fungal DNA with ash die-back specific primers yielding an amplicon of the expected size in all inoculated, but no control, tissues (Fig. 2E).

### *H. fraxineus* associates with ash seedling roots when germinated on infected soil and subsequently inside the leaves

**Figure 3:**
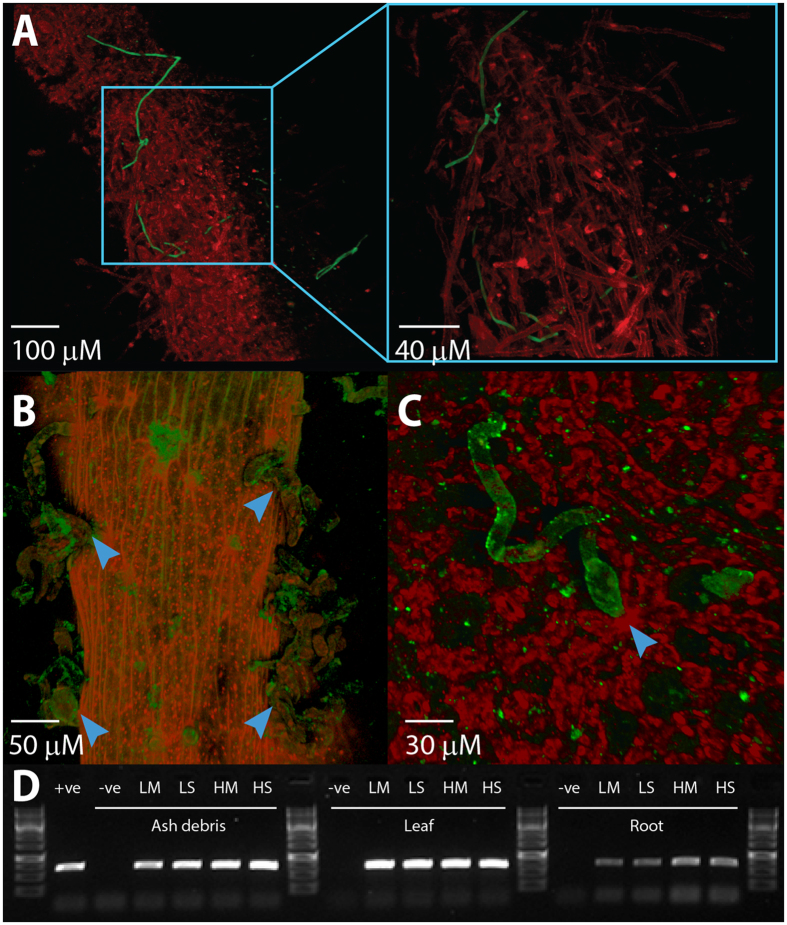
Confocal microscopy images showing *H. fraxineus* on ash material grown on soil inoculated with asexual spores or mycelia; fungus stained with FITC-WGA (green) and ash tissue stained with PI (red). (**A**) *H. fraxineus* hyphae among the root hairs of ash seedlings (enlarged view at right); (**B**) *H. fraxineus* emerging from the leaf vascular tissue and (**C**) emerging from leaf epidermis (blue arrowheads). (**D**) PCR detection of *H. fraxineus* in root, leaf and ash debris samples from inoculated soil (LS, low spores; HS, high spores; M, mycelium; −ve, uninfected; +ve, control *H. fraxineus* DNA from culture). Three independent replicates are shown.

To test the hypotheses that asexual spores of *H. fraxineus* germinate in soil and infect ash seedlings *via* the roots, or can colonise abscised leaves and seed coats (ash debris), we inoculated soil with *H. fraxineus* spore suspensions or with mycelium at two inoculum densities each. Ash seeds were sown in these soils, as well as in non-inoculated soil, and seedlings were harvested twelve weeks later. Leaves and roots of these ash seedlings were stained and visualised by confocal microscopy. This revealed the presence of hyphae among, and wrapped around, the root hair cells, but apparently not within the root (Fig. 3A). The leaves, however, showed bundles of, and individual, hyphae emerging mainly from the vasculature (Fig. 3B,C). The fungus thus appeared to have invaded the aerial tissues of the ash seedlings *via* the roots from soil inoculum. DNA was therefore extracted from three independent samples from each treatment, each consisting of the roots or leaves of five seedlings. DNA was also extracted from samples of ash debris taken from the surface of the soil of each treatment. *H. fraxineus* specific PCR revealed the presence of the fungus in spore-inoculated and mycelial-inoculated treatment in each sample type (Fig. 3D), indicating that the fungus had been able to associate with the ash and migrate up the plant regardless of the form of the initial inoculum.

### *H. fraxineus* grows as a soil fungus in the absence of ash tissue

**Figure 4:**
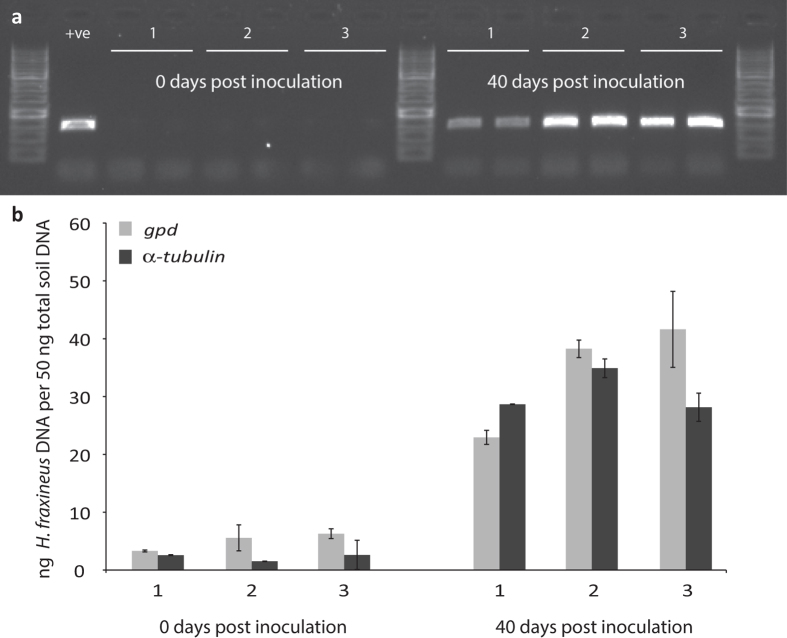
(**a**) PCR detection of *H. fraxineus* in samples of inoculated soil on Day 0 (left) and Day 40 (right). +ve, control *H. fraxineus* DNA from culture. Three independent soil replicates were used (labelled 1, 2 and 3, and two technical replicates of the PCR were performed for each); (**b**) qPCR of same soil DNA samples, showing estimated *H. fraxineus* DNA concentrations (in ng/50 ng total soil DNA) based on each of two genes. Both genes show a significant increase in the amount of *H. fraxineus* DNA over the 40 day period in all three samples, with no significant difference between the two genes.

Finally, we investigated whether *H. fraxineus* survives and grows in the soil in the absence of ash. We inoculated three petri dishes of soil with *H. fraxineus.* Three samples were taken, each pooled from five random points in each dish on the day of inoculation. This was repeated after forty days maintenance of the dishes at high humidity, at 20 °C. *H. fraxineus* specific PCR primers did not detect fungus in the day 0 samples, but returned strong bands of the expected size after incubation for 40 days (Fig. 4a). To confirm this result, we carried out qPCR on the same DNA, using primers designed to amplify the *glyceraldehyde-3-phosphate dehydrogenase (gpd*) and *α*-*tubulin* genes of *H. fraxineus* (Fig. 4b). Both genes showed a significant increase in *H. fraxineus* DNA over the 40 day period (p = 0.015 (*gpd*) and 0.0004 (*α*-*tubulin*); *t-*tests with Bonferroni correction). The average fold-increase for *gpd* across the three samples was 6.81x, while for *α*-*tubulin* this was 14.96x; the two average fold-increases were not, however, significantly different (p = 0.11; *t-*test). This finding demonstrates fungal survival and an increase in fungal biomass in ash-free soil.

## Discussion

In this work, we have demonstrated that the conidia of *H. fraxineus* are capable of germination, albeit at low frequency. This germination takes place on both hydrophobic glass slides overlaid with ash waxes, and ash leaves, where it can lead to infection. *H. fraxineus* can colonise soil and ash debris and is able to colonise ash seedlings *via* association with the root, followed by infection of the aerial tissue. This seedling infection appears to be systemic, based on PCR detection assays and microscopy, although the course of infection may vary in saplings and mature trees. Such soil-borne infection could be responsible for lesions seen at the root collar of saplings or mature trees. The low germination rate and viability of conidia and germlings is consistent with the behaviour of microconidia in *M. oryzae*^34^.

**Figure 5:**
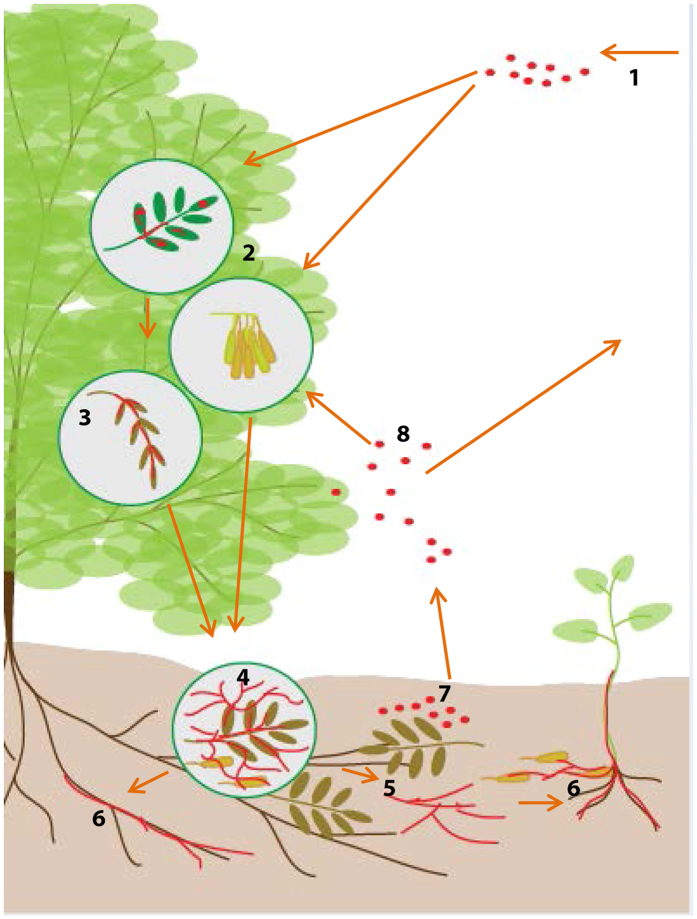
Model outlining the possible roles of asexual spores in ash infection by *H. fraxineus*. 1. Spores arrive in the environment of a susceptible ash tree (e.g. via wind). 2. Spores germinate on leaves and on ash seed cases. 3. Leaves become infected, with the fungus growing over the surface and forming internal structures, which may be fruiting bodies, amplifying the inoculum. 4. Infected leaves are abscised along with the ripe seeds; the fungus continues to propagate on leaf litter, seed cases and other debris, as well as in the soil itself (5). 6. From the soil, mycelium/germinated spores can invade the roots of mature trees and seedlings. 7. Sporulation, including sexual sporulation if strains of opposite mating types are present, may then occur in the leaf litter, releasing inoculum to re-infect the original host tree and others nearby (8).

Based on these data, we have developed a model for the role of conidia in the lifecycle of the pathogen, a schematic of which is provided in Fig. 5. In this model, conidia germinate on leaves, causing necrosis, and infected leaves drop to the forest floor. If the structures seen within the leaves are, indeed, fruiting bodies, then amplification of the inoculum in the absence of a mating partner may occur. Even without new spores, existing mycelium proliferates in the soil, on fallen leaves and on seed coats, demonstrated here. Finally, this mycelium encounters ash roots, tracking along them to colonise and infect a new ash tree.

This model has implications for the way in which we consider host infection and disease spread by *H. fraxineus.* Firstly, we have, until now, considered the potential for infection based on ascospore numbers alone, ignoring the role played by the asexual spores. Despite their low germination rates, conidia might contribute significantly to the rapid spread of infection. Aerial spread of *H. fraxineus* conidia has been proposed (Stenlid pers. com.), but not proven. The scanning electron micrograph images of sporulating plates of *H. fraxineus* show a contiguous ‘sea’ of spores, including globular and chain-like structures, which supports reports that conidia are sticky^12^. It would be interesting to determine whether extracellular mucilage is present, and if it is removed through rain-splash or dehydration, allowing conidia to separate and become airborne. Alternatively, sticky mucilage could assist conidial dispersal by insect vectors, which could explain the genetic differences between upland and lowland populations found by Kraj *et al*.^22^. Either of these scenarios would mean that conidia play a role in inter-population gene flow and pathogen range expansion.

Secondly, this model provides a route for systemic infection of ash in the absence of *H. fraxineus* strains of opposite mating types. This increases the risk of spreading ash dieback disease *via* transfer of small quantities of spores or mycelium.

However, given the high intra-population genetic diversity, the greatest significance of the findings reported here lies in the potential for asexual sporulation in leaves and elsewhere. Conidia produced on infected leaves may be spread throughout the canopy by rain-splash, as in other plant pathogenic fungi which exude spores in extracellular mucilage (e.g. *Zymoseptoria, Fusarium*). Thus, strains originating from ascospore infection may spread throughout a stand of ash trees, and, if multiple strains are present, they may each be dispersed throughout the stand. In the leaf litter, the fungus can also grow on ash debris and in soil. This could vastly increase the numbers of conidia available on the forest floor to act as spermatia. Thus, this process could increase the spread and availability of parent strains in a small area, and may underpin the usually high levels of outcrossing which characterise *H. fraxineus*^14^,^19^.

These high levels of outcrossing and inter-population gene flow in *H. fraxineus* are cause for concern when considering the risks to forest ecosystems and forestry, as they indicate that any beneficial genotype arising through mutation, introgression from related fungi or from horizontal gene transfer (HGT) may spread rapidly within and between populations. This gives *H. fraxineus* a high evolutionary potential, even in comparison with other emerging infectious fungal diseases. As a result, this pathogen is unlikely to be long confined geographically by sub-optimal temperatures or competitors, and its prospects of survival and spread in the face of climate change are very fair. High evolutionary potential also raises the spectre of increased risk of the successful transition of *H. fraxineus* onto new hosts. The acquisition of a virulence gene allowing the fungus to attack additional hosts would be followed by the rapid spread of this new pathogenicity throughout the fungus’ range.

Studies indicate that whilst *H. fraxineus* is unable to infect species of the Oleaceae outwith the genus *Fraxinus*^38^, it can grow on leaf-extract agar made from plants of various genera within this family, including the economically important *Olea europaea*^39^. *H. fraxineus* is already pathogenic towards a number of ash species in addition to its main host, *F. excelsior*^2^,^4^,^26^,^40^,^41^. Although *H. fraxineus* is not able to hybridise with *H. albidus*^42^, there are known to be sympatric species of *Hymenoscyphus*^43^,^44^,^45^ which may be potential hybridisation partners from which virulence and pathogenicity genes might be acquired; some of these strains also produce conidia. Worryingly, Japanese strains of *H. fraxineus* are more virulent on both *F. excelsior* and *F. pennsylvanica* than are European strains^40^, indicating that the global *H. fraxineus* population already contains additional virulence genes. In a world of globalised trade, travel and transport, it is only a matter of time until more virulent strains reach Europe. These risks mean that a full understanding of the breeding strategies of *H. fraxineus* and its close relatives is needed to protect ash in Europe.

In conclusion, the previously overlooked potential of *H. fraxineus* conidia to germinate and cause infection may underpin the success of the pathogen by facilitating sexual reproduction, increasing gene flow between sympatric strains. This may expediate the rapid evolution of *H. fraxineus*, allowing this pathogen to attack *F. excelsior* and other ash species, and increasing the risk of its jumping onto additional *Fraxinus* or Oleaceae species. This rapid evolution also allows *H. fraxineus* to colonise new areas and adapt to new climates.

## Additional Information

**How to cite this article**: Fones, H. N. *et al*. A role for the asexual spores in infection of *Fraxinus excelsior* by the ash-dieback fungus *Hymenoscyphus fraxineus.Sci. Rep.* **6**, 34638; doi: 10.1038/srep34638 (2016).
